# The effect of pre-existing health conditions on the cost of recovery from road traffic injury: insights from data linkage of medicare and compensable injury claims in Victoria, Australia

**DOI:** 10.1186/s12913-016-1386-6

**Published:** 2016-04-29

**Authors:** Behrooz Hassani-Mahmooei, Janneke Berecki-Gisolf, Youjin Hahn, Roderick J. McClure

**Affiliations:** Institute for Safety, Compensation and Recovery Research, Monash University, Melbourne, Australia; Monash Injury Research Institute, Monash University, Melbourne, Australia; Department of Economics, Yonsei University, Seoul, South Korea; Department of Economics, Monash University, Melbourne, Australia; Harvard Injury Control Research Centre, Harvard School of Population Health, Boston, USA

**Keywords:** Pre-existing conditions, Health service use, Recovery, Traffic accident, Injury, Australia

## Abstract

**Background:**

Comorbidity is known to affect length of hospital stay and mortality after trauma but less is known about its impact on recovery beyond the immediate post-accident care period. The aim of this study was to investigate the role of pre-existing health conditions in the cost of recovery from road traffic injury using health service use records for 1 year before and after the injury.

**Methods:**

Individuals who claimed Transport Accident Commission (TAC) compensation for a non-catastrophic injury that occurred between 2010 and 2012 in Victoria, Australia and who provided consent for Pharmaceutical Benefits Scheme (PBS) and Medicare Benefits Schedule (MBS) linkage were included (*n* = 738) in the analysis. PBS and MBS records dating from 12 months prior to injury were provided by the Department of Human Services (Canberra, Australia). Pre-injury use of health service items and pharmaceuticals were considered to indicate pre-existing health condition. Bayesian Model Averaging techniques were used to identify the items that were most strongly correlated with recovery cost. Multivariate regression models were used to determine the impact of these items on the cost of injury recovery in terms of compensated ambulance, hospital, medical, and overall claim cost.

**Results:**

Out of the 738 study participants, 688 used at least one medical item (total of 15,625 items) and 427 used at least one pharmaceutical item (total of 9846). The total health service cost of recovery was $10,115,714. The results show that while pre-existing conditions did not have any significant impact on the total cost of recovery, categorical costs were affected: e.g. on average, for every anaesthetic in the year before the accident, hospital cost of recovery increased by 24 % [95 % CI: 13, 36 %] and for each pathological test related to established diabetes, hospital cost increased by $10,407 [5466.78, 15346.28]. For medical costs, each anaesthetic led to $258 higher cost [174.16, 341.16] and every prescription of drugs used in diabetes increased the cost by 8 % [5, 11 %].

**Conclusions:**

Services related to pre-existing conditions, mainly chronic and surgery-related, are likely to increase certain components of cost of recovery after road traffic trauma but pre-existing physical health has little impact on the overall recovery costs.

## Background

Road traffic crashes are one of the most common external causes of injury leading to hospitalisation among young people in high income countries throughout the world, including Australia [3], New Zealand [23], Europe [10] and the United States [22, 38]. In the United States, the economic cost of motor vehicle accidents was estimated to be close $277 billion in 2010 equivalent to 1.9 % of the Gross Domestic Product (GDP) [4]. The social costs of road traffic crash injury in Australia in 2006 were estimated to be $18,000 million, which is equal to about 1.7 % of the GDP. In that year, 1602 people died and more than 31,000 injured in road traffic crashes. Sixty seven percent of these injured people stayed one or more nights at the hospital and 4619 people suffered disability, from which 1270 people had “severe or profound limitation”. Cost of these injuries were approximately $2.4 million per fatality, approximately $214,000 per hospitalised injury (including disability-related costs), and approximately $2200 per non-hospitalised injury” [3].

Several studies have found that health conditions (or comorbidity) that existed before the crash event (e.g. diabetes, heart disease) can be predictive of mortality after serious injury. This has been demonstrated in trauma patients in the acute care setting [20, 21, 30, 32, 35] and among older adults aged ≥65 years admitted to emergency departments for home or road injury [7]. Pre-existing health conditions have also been shown to predict morbidity after trauma. In particular, such correlations have been found for a number of chronic comorbid conditions with respect to its impact on post injury length of hospital stay. Disability at 12 months post-injury has also been found to be affected by pre-injury comorbidity among orthopaedic injury patients [12].

While prior literature suggests that pre-existing health conditions have a deleterious effect on post injury outcomes, the nature and extent of this relationship is not well documented. Research on the impact of comorbidity on trauma outcomes has mainly been conducted from a clinical perspective, with a focus on improving triaging and trauma management procedures. Minor injury that does not require hospitalisation is not captured in trauma-centre based research and little is known about the relationship between pre-existing health conditions and outcomes of minor injury. One of the main advantages in our study is that we use comprehensive linkage dataset on medical and pharmaceutical usage. Thus, the analysis is not limited to hospitalised patients and their services used in hospital.

While often overlooked because of their minor injury status, people with non-hospitalised injuries frequently have delayed recovery. Recent studies have shown that non-hospitalised injuries account for the majority of the injury incidence and the majority of the injury burden [16]. Of the compensable road traffic injuries in Victoria, Australia, approximately 70 % of injury claims are for injuries that do not require hospitalisation, and little is known about the impact of comorbidity on cost health service use after injury in this group. Given the minor nature of many of the non-hospitalised injuries, much of the long term burden in this group may be due to the adverse impact of pre-existing conditions rather than being mainly the consequence of the injury itself. Increased levels of post injury care and additional tests and procedures may be indicated in people with comorbidity and this may independently increase the post injury economic costs related to care for this group.

If there is a relationship between pre-existing health conditions and cost of road traffic crash injuries, then we could expect over the next 20 years to see a dramatic escalation of these costs and substantial challenges in providing health services. The population is ageing as the baby boom generation (birth years 1946–1965) is reaching retirement age. The baby boom generation is more likely to drive a car than the generations before them, and baby boomers are likely to continue driving as they age. The road user population can therefore be expected to age more rapidly than the general population [28]. Chronic conditions are likely to be prevalent in an ageing population and have previously been associated with trauma outcomes; these include diabetes mellitus [13, 21] and cardiovascular disease [20, 21]. As the road user population ages, the prevalence of chronic disease among road users, and among trauma victims, can be expected to increase. Insight into the effect of pre-existing chronic disease on injury recovery, and the cost of recovery, is essential if the larger health care system (of which the road transport injury compensation schemes are a part) is to anticipate and accommodate a potentially increasing economic and social burden.

With the purpose of helping prepare health and disability care industries meet their impending challenges, we undertook a study that aimed to quantify the effect of pre-existing health conditions on the cost of recovery from road traffic injury. Consistent with the study purpose, we undertook economic analyses from a payer perspective and used administrative health care system and traffic crash compensation scheme data as inputs to our models. The chosen study region, the state of Victoria in Australia, has a universal population coverage of health service delivery, as well as pharmaceutical and road traffic injury insurance coverage. Using a comprehensive dataset obtained by linking the three relevant administrative databases of medical insurance, pharmaceutical benefits, and traffic injury coverage, this study offers a unique opportunity to evaluate the role of comorbidity in injury recovery.

## Methods

### Setting

Victoria is a state in south east of Australia with a population of 5,821,300 (March 2014). The Transport Accident Commission (TAC) is a state-government organisation responsible for paying the cost of treatment rehabilitation services, disability services, income assistance, travel and household support services to people injured in traffic accident in the state of Victoria regardless of whether the client was at fault. It is a population based scheme funded from annual car registration payments by Victorian motorists. The treatment payments include the categories such as ambulance, hospital and medical services, pharmacy items, and equipment. There is AUD$623 (as of 2014) medical excess for these categories excluding the ambulance and hospital services. TAC also pays for other services such as child care, loss of earning, as well as lump sum compensation such as impairment benefit.

Medicare Benefits Schedule (MBS) is an Australian Federal Government scheme that “provides access to medical and hospital services for all Australian residents and certain categories of visitors to Australia. Medicare benefits are claimable only for ‘clinically relevant’ services rendered by an appropriate health practitioner” [17]. Pharmaceutical Benefits Scheme (PBS), (also and Australian Federal Government initiative) “gives all Australian residents and eligible overseas visitors access to prescription medicine in a way that is affordable, reliable and timely”. MBS and PBS are administered by the Department of Human Services, Canberra, Australia.

### Study design

A consented data linkage study was undertaken, with the inception cohort being a subset of persons with compensable road traffic injury. Research data was obtained by linking of the TAC claims data of consenting study participants, with health data from the MBS and PBS provided by the Department of Human Services (Canberra, Australia) to obtain relevant information about the cohort for 12 months prior to and 12 months following the date of their injury. The study aim was addressed by quantifying the association between the pre injury health (determined from health service and pharmaceutical use) and post injury claim cost, controlling for confounders.

### Study population

People registered in the Transport Accident Commission claims database with an incident injury from a road crash in Victoria dating between 17/07/2011 and 22/07/2012 were invited to participate. Potential study participants were excluded if they had (1) an acquired brain injury; (2) Glasgow Coma Scale is less than 9;(3) length of Post Traumatic Amnesia greater than 6 days; or (4) one or more of the following injuries: paraplegia, quadriplegia, amputation above elbow or above knee, severe burns (full thickness, approximately >30 % whole body) and blindness due to nerve severance. Also, claimants who were involved in an accident where there was a fatality were excluded.

### Ethics, process and study approval

The study was first approved by the Monash University Human Research Ethics Committee (MUHREC): Project number CF12/0875 – 2012000398 ‘After MUHREC approval, the study was also evaluated by the External Request Evaluation Committee at the Department of Human Services in Canberra.

Study invitations were mailed to participants in three batches: a pilot mail-out to test the study logistics, followed by two main mail-outs. For the final (and largest) mail-out, the initial study invitation was followed by a reminder package including a reminder letter, a new set of forms (explanatory statement and consent form) and a reply paid envelope addressed to Monash University.

Contact details of eligible participants were provided to the printer by the TAC. Reply paid envelopes were provided by the researchers at the Monash Injury Research Institute (MIRI). Study invitations were printed and posted by the printer: name, address, date of birth and study linkage dates (based on the claim date) were pre-filled on the Medicare consent form. The researchers at MIRI were not provided with contact details of the selected sample; they only received the signed and posted consent forms of participants providing their consent. The TAC was not informed about participation of individual clients. The Department of Human Services provided the Medicare and Pharmaceutical Benefits Scheme records starting 12 months prior to, through to 18 months after the claim onset, of participants who provided consent. This dataset was linked to TAC claims and payments records from the Compensation Research Database at the Institute for Safety, Compensation and Recovery Research (ISCRR). For this analysis, the linked dataset did not contain personal identifiers and was restricted to pre-injury MBS and PBS records only, linked with TAC payments that occurred in the 12 months following the injury (i.e. a 12-months follow-up).

### Variables and data sources

#### Participant status, injury, age and sex

Participant information was obtained from the TAC claims records: this includes gender, age, socio-economic indicators for clients’ residential areas, role in the accident (e.g. driver, cyclist, motor-cyclist, pedestrian, and passenger), number of vehicles involved in the accident, location of accident (metropolitan, country or interstate), and type of injury such as musculoskeletal (e.g. contusion), orthopaedic (e.g. fractures and dislocations), brain injury and internal injury.

#### Pre-existing health conditions

Pre-injury health conditions (comorbidity) were inferred from information obtained from both the Medicare Benefits Schedule (MBS) and Pharmaceutical Benefits Scheme (PBS) records for 12 month period immediately prior to the injury. Although health service use and pharmaceutical records do not provide clinically validated disease diagnoses, overall service use reflects the intensity of health care utilisation prior to injury. Indications of the prevalence of these conditions were based on the use of pharmaceuticals as well as specific diagnostic and therapeutic procedures.

#### Cost of injury

In this study, the cost of injury refers to the direct cost of any health service use which is compensated by the TAC. It includes the cost of ambulance, hospital and medical services and is measured in Australian dollars. The cost of injury was determined from injury compensation payment records over 12 months following the injury. The dataset holds a record of every payment made by the TAC to a client or provider. Progression from short-term to long-term costs was considered, by making the distinction between the 3, 6 and 12 months cost of recovery. Taking into account the biased distribution of service cost among participants coming from heavy service use by a small number of participants, both original and log values of service count are used.

### Data management and analysis

Data from the MBS and PBS were used to create markers of pre-existing health in the following manner.

The 6000 different items of service available in MBS data were used to derive information about underlying health of the participants. The Bayesian Model Averaging (BMA) was used to select the most efficient MBS items in the dataset to represent comorbidities for subsequent inclusion in the final analytic multivariate regression models. The BMA is commonly accepted approach when there is model uncertainty, and widely used in several disciplines such as economics, political science, ecology, environmental science [5, 11, 26, 27, 37]. To ensure that the multicollinearity across included variables did not affect measured uncertainty within the MBA models, dilution method was used as suggested by Durlauf et al. [9], which essentially puts a greater penalty to models with highly collinear variables.

To categorise treatment for pre-existing health conditions, PBS codes were provided with corresponding Anatomical Therapeutic Chemical (ATC) classification system codes [19]. The ATC coding has five levels but considering the level of data availability, the top two levels only were used in the analysis.

## Results

### Sample characteristics

In total 10,998 potential study participants were invited to participate. Informed consent for inclusion in the study with both MBS and PBS data linkage was obtained from 738 TAC clients (i.e.7 %). Comparing the participating sample with the invited sample in terms of claim information, women were more likely to participate than men (RR = 1.36, 95 % CI: 1.17, 1.59). Study participants were older with a median age of 50 years (25^th^ and 75^th^ percentiles of 35, 63); compared to a median age of 39 years (25^th^ and 75^th^ percentiles of 26, 54) years in the mail-out sample overall. Clients with claims for musculoskeletal injuries were less likely to participate than those with orthopaedic (RR = 1.59, 95 % CI: 1.27, 1.98) or other injuries (RR = 1.57, 95 % CI: 1.24, 1.98). All the claimants were followed for at least 1 year, though 33 % did not have any compensation received beyond the day of accident, 7 % received payments only between 1 and 7 days after the accident and 20 % were compensated only between one and 26 weeks after the accident.

### MBS data

Out of the 738 study participants, 688 used at least one MBS item in the year prior to their accident. The total number of pre-injury service items was 15,625. Following MBS categorisation, all medical services were grouped under eight main categories and 91 groups. Overall, 85 % of the services were categorized as attendance or pathology services. While the greatest share of the overall cost belongs to attendances, due to their high usage, the most expensive individual service items were surgeries, operations and organ replacements. From approximately 6000 different service items on the MBS, study participants used only 751 different services across the eight categories. For many of these services, the number of study participants who had used the service was not sufficient to support reliable estimates and these services were removed from further analyses. Services with usage patterns that were logically correlated (e.g., skin biopsy followed by pathology service) were identified and the relevant collinear items removed. After removing the factors with low number of observations, three categories, 52 groups/subgroups and 66 items were included in the final MBS dataset.

### PBS data

The PBS dataset contained 9846 records by 427 study participants (58 %). Among more than 550 items that were listed, rosuvastatin (a statin) and atenolol (a beta blocker) were the most frequently occurring items.

The most frequently occurring categories in the second level were C09: Agents acting on the renin-angiotensin system, C10: Lipid Modifying Agents, N02: Analgesics, N06: Psychoanaleptics, and finally A02: Drugs for Acid-related Disorders. After removing highly correlated items, 20 PBS variables remained.

### Transport accident compensation costs by category

From the total of 14.3 million dollars paid for injury-related services among study participants in the 12 months post-injury, more than 60 % was paid for hospital and medical costs (Table 1).

**Table 1:** Cost categories and their share in total cost (in Australian Dollars)

Service group	Count of items	%	Avg. cost of item	Sum of cost	%
Ambulance/Road accident rescue	848	1.34 %	$1423.29	$ 1,206,954	8.45 %
Hospital	1287	2.03 %	$4880.94	$ 6,281,771	43.96 %
Medical	21,818	34.47 %	$120.40	$ 2,626,989	18.38 %
Total cost of health-related services				$ 10,115,714	70.79 %
Total cost of non-health services				$ 4,173,459	29.21 %
Total cost of services				$ 14,289,173	100 %

For each of the cost categories, the data was further divided into four time ranges: 1 month, 3 months, 6 months and 1 year. For some cost categories, the majority of expenses were clustered in the first month post-accident (e.g. ambulance services) while other expenses continued beyond the first 6 months (e.g. medical services). The mean cost in the first month of non-hospitalised clients was 4538 [95 % CI: 3424, 5652] compared to 32,044 [95 % CI: 27,526, 36,563] for hospitalised clients. The 3-month cost increased to 5288 [95 % CI: 3951, 6625] for non-hospitalised and 40,780 [95 % CI: 34,627, 46,933] for hospitalised clients; and at 1 year the costs increased to 6970 [95 % CI: 5184, 8757] and 54,063 [95 % CI: 45,472, 62,654], respectively (all in Australian Dollars based on 2012 prices).

### Cost of injury recovery: ambulance

Participants who used pre-injury diabetes medication (ATC code A10: Drugs used in Diabetes) had $108.13 higher ambulance costs compared with those who did not, for every prescription received in the year before the accident [95 % CI: 75.10, 141.15]. Since the number of prescriptions of diabetes medications for participants who have received at least one prescription was distributed with the mean of 7.7 prescriptions per person and standard deviation of 5.9, it is expected that each participant using diabetes drugs had, on average, approximately $831 higher ambulance costs [95 % CI: 194, 1470].

### Cost of injury recovery: hospital

Table 2 presents the pre-injury service items that were statistically associated with post-injury hospital costs. MBS Item 66512, *Five or more tests from Quantitation in serum, plasma, urine or other body fluid*, is considered as the highest ladder pathological item considered mainly as a cost item for chronic conditions [1, 24, 33]. Three other pre-injury pathological services were associated with post-injury hospital costs indicating diabetes, hepatitis and thyroids.

**Table 2:** The pre-injury service items that are statistically associated with the post-injury hospital cost (in Australian Dollars)

Variable	Coefficient^c^
Outcome Variable: Post-injury Hospital Cost^a^	
Five or more tests from Quantitation in serum, plasma, urine or other body fluid (MBS Item 66500)	$3799 [$2337, $5261]
Quantitation of glycosylated haemoglobin performed in the management of established diabetes (MBS Item 66551)	$10,406.52 [$5467, $15,346]
Outcome Variable: Post-injury Hospital Cost, Logged^b^	
Anaesthetist, pre-anaesthesia consultation (MBS Sub Group T6-1)	0.24 [0.13, 0.36]
Two tests from tests for hepatitis antigen or antibodies to determine immune status or viral carriage following exposure or vaccination to Hepatitis A, Hepatitis B, Hepatitis C or Hepatitis D (MBS Item 69475)	0.65 [0.37, 0.92]
Thyroid function tests (MBS Item 66719)	0.37 [0.06, 0.69]

^**a**^The coefficient indicates the average change in the category cost for every extra item used from the services listed based on a linear model. ^b^The coefficient indicates the average percentage change in the category cost for every extra item used from the services listed based on a nonlinear model. ^c^[95 % confidence interval]

### Cost of injury recovery: medical

Compensated medical services include consultations (general practitioners, physicians, specialist, etc.), hospital outpatient medical services, pathology and radiology. Table 3 presents the main pre-injury factors found to be associated with greater overall medical recovery costs.

**Table 3:** The pre-injury service and items which have significant impact on the medical costs of recovery after road traffic injury (in Australian Dollars)

Variable	Coefficient^c^
Outcome Variable: Post-injury Medical Cost^a^	
Cardiovascular diagnostics and investigation (MBS Sub Group D1-6)	$76.44 [$54.27,$98.62]
Initiation of management of anaesthesia (MBS Sub Group T10-6)	$257.65 [$174.16,$341.16]
Thyroid function tests (MBS Item 66719)	$356.28 [$258.79, $453.78]
Prothrombin time test (MBS Item 65120)	$26.77 [$15.02, $38.53]
Vascular Ultrasound (MBS Sub Group I1-3)	$359.79 [$273.77, $445.82]
Radiographic examination of head (MBS Sub Group I3-3)	$301.92 [$186.58, $417.28]
Drug for Anti-inflammatory and Anti-rheumatic Products (ATC Code M01)	$49.75 [$35.4, $64.12]
Drugs for Sensory Organs (ATC Code S01)	$31.48 [$23.04, $39.93]
Outcome Variable: Post-injury Medical Cost, Logged^b^	
Drugs used in diabetes (ATC Code A10)	0.08 [0.05, 0.11]

^a^The coefficient indicates the average change in the category cost for every extra item used from the services listed based on a linear model. ^b^The coefficient indicates the average percentage change in the category cost for every extra item used from the services listed based on a nonlinear model. ^c^[95 % confidence interval]

Participants who used anti-inflammatory and anti-rheumatic drugs (mainly Meloxicam, Celecoxib, and Diclofenac(L7), ophthalmological (L8) and diabetes medications had, on average, higher medical recovery costs. For example, in the first year after the injury, each participant who used Anti-inflammatory and Anti-rheumatic Products medication cost an extra $49.75 [95 % CI: 35.4, 64.12] compared to those who did not. In the 12 months after the accident, patients who used diabetes medications had close to 8 % higher medical consultations cost than those not on diabetes medication.

Moreover, patients with electrocardiography in the year before their injury were more likely to have medical costs, in particular medical consultations. Surgery (identified through anaesthetic services and as well as specific surgery procedure Medicare items) affects medical costs as well. Short-term medical costs were also affected by pathological tests, namely thyroid function tests and prothrombin time test. While the coefficient of Thyroid test was much larger than the Prothrombin test, it should be noted that the average number of Thyroid tests was two per person, compared to 17 per person for Prothrombin time.

### Total cost

None of the pre-injury MBS or PBS service items included in the analysis was significantly associated with the total injury compensation cost in either original or log values.

## Discussion

This study has two main findings. The first is that pre-injury health service use and corresponding health conditions were not associated with the overall cost of recovery in our sample. Most estimates of the cost of injury derived from administrative data sets are unable to identify the pre-injury physical health of the injured sample due to data limitation [25, 31]. The findings from this study provide some confidence that the validity of these cost-of-injury estimates is not significantly threatened by this lack of pre-existing physical health data.

The second main finding of the study is heterogeneous effect of pre-existing conditions on the post injury outcomes. Not all costs of recovery were affected by the pre-existing conditions; the effect was mainly restricted to health service costs. Diabetes and cardiovascular conditions had the highest level of impact on the subcategories of post injury recovery costs such as medical costs. Receiving surgery-related therapeutic services such as general surgeries or anaesthesia in the year before the accident were also associated with increases in some cost categories such as post-injury hospital costs.

While the literature on the cost outcomes of patients with traffic accidents Our findings are in line with earlier studies indicating the increased risk of adverse outcomes in patients with diabetes [2, 8, 18, 30], cardiovascular conditions [20, 30, 34, 35] and surgeries [29, 36]. The significant impact of having diabetes on the cost of recovery can be explained. First, patients with diabetes are likely to need monitoring of their diabetes during the acute phase of the injury, and second, they are most likely to develop complications such as delayed healing of wounds and infections. The observed relationship between surgery in the year before injury and increased cost of injury recovery may be due to the lack of resilience that is manifest during the post-surgery convalescence.

Our findings also highlight that the impact of pre-existing chronic conditions on recovery outcomes is not fully captured by easily observable demographic characteristics such as age. Prevalence of pre-existing chronic conditions is likely to increase with age [2, 30, 35] and the average age of drivers is increasing [6, 14]. Findings from earlier studies suggest that older individuals’ sustained injuries appear more serious [15] and comorbidities and complications among older patients are more likely to lead to poorer hospital outcomes than younger counterparts [6]. As we find that certain pre-injury health conditions matter on recovery outcomes even after controlling for age, the increased comorbidities and medical complications observed among older individuals may at least partly be due to these specific conditions not necessarily due to age per se.

The main strength of this study was the use of health service records, before and after the injury to identify the links between pre-injury comorbidities and post-injury recovery. Considering the comprehensiveness of the three datasets, MBS, PBS and TAC, the study was able to provide more robust conclusions than studies that use self-stated indicators, particularly self-reported pre-injury health.

One of the weaknesses of this study is the low rate of consent among potential study participants. There is a participation bias with relative overrepresentation of older persons in the study. Pharmaceutical and service use is likely to be higher in the study sample than in the underlying population. The prevalence of mental health conditions, inferred from service and medication use, is likely to be overestimated in this study; however, interval validity of the correlations between mental health conditions and the cost of recovery are unlikely to be affected.

Another weakness of the study is that Medicare items were used as indicators of health conditions rather than specific diagnosis variables. Medicare items include many “investigative” services which may not necessarily equate to morbidity. Furthermore, untreated disease could not be identified using these data.

We used Bayesian Model Averaging to achieve robust variables based on the Medicare and pharmaceutical data, but this is not as effective as linking these data to actual diagnostic data. Further research could include a validation study of liked Medicare and pharmaceutical records linked with hospital admission records including ICD10 comorbidity codes. This may also provide an estimate of the prevalence of untreated disease, which may be picked up in the ICD10 codes but not in the Medicare or pharmaceutical data.

And finally, many of the potentially informative pre-injury health service items could not be included in the analysis because they were not frequent enough in the sample. The analysis can also be improved by using a larger sample and by taking into account longer pre-injury and post-injury time periods.

Future research should focus on analysing linked data over longer pre- and post-injury durations as well as including other datasets such as hospital admissions in the linkage process.

## Conclusions

This study shows that the services related to pre-existing conditions, mainly chronic and surgery-related, are likely to increase certain components of road trauma recovery cost, but that overall pre-injury health service use has little impact on the overall injury recovery costs. Also, the impact of pre-existing chronic conditions on cost of recovery cannot be fully attributed to age. Our findings are expected to help the compensation agencies such as TAC since better understanding of the drivers of traffic accident compensation costs is a key step in: 1) delivering a fairer and further optimised system of providing benefits to compensation and insurance clients through better allocation of financial resources, 2) better internal management of claims and efficient allocation of physical and human resources and therefore greater client satisfaction, and 3) ensuring faster recovery, return to work and more effective as well as efficient service provision.

### Ethics statement

The study was first approved by the Monash University Human Research Ethics Committee (MUHREC): Project number CF12/0875 – 2012000398 ‘After MUHREC approval, the study was also evaluated by the External Request Evaluation Committee at the Department of Human Services in Canberra.

### Availability of data and materials

The data cannot be publicly shared due to ethical considerations but it can be requested from JBG, if compliant with ethics and aligned with the original study purpose as described in the participant explanatory statement. The statistical code can be shared if BHM is contacted.

## Abbreviations

TAC: transport accident commission
PBS: pharmaceutical benefits scheme
MBS: medicare benefits schedule
MUHREC: Monash University Human Research Ethics Committee
MIRI: Monash Injury Research Institute
ISCRR: Institute for Safety, Compensation and Recovery Research
BMA: Bayesian model averaging
ATC: anatomical therapeutic chemical
